# Myocardial perfusion reserve is low in heart transplant patients and is related to exercise capacity

**DOI:** 10.1111/cpf.70082

**Published:** 2026-07-16

**Authors:** Kristian Dimovski, Grunde Gjesdal, Katarina Steding‐Ehrenborg, Robert Jablonowski, Hui Xue, Peter Kellman, Oscar Braun, Håkan Arheden, Henrik Engblom

**Affiliations:** ^1^ Department of Clinical Physiology, Department of Clinical Sciences Lund Lund University, Skåne University Hospital Lund Sweden; ^2^ Cardiology, Department of Clinical Sciences Lund Lund University, Skåne University Hospital Lund Sweden; ^3^ National Heart, Lung, and Blood Institute, National Institutes of Health, DHHS Bethesda Maryland USA

**Keywords:** quantitative CMR, myocardial perfusion, microvascular dysfunction, CPET, cardiac transplant

## Abstract

**Aims:**

Heart transplant (HTx) patients are known to be at risk of coronary microvascular dysfunction as well as having decreased exercise capacity. The aim of this study was to investigate if microvascular function in HTx patients differs from healthy controls and is related to objective measures of exercise capacity.

**Methods and Results:**

Twenty‐nine HTx patients (51 ± 15 years, 34% women) and 26 healthy controls (57 ± 10 years, 38% women) underwent CMR. Quantitative myocardial perfusion maps were acquired using single‐bolus (0.05 mmol/kg), dual‐sequence perfusion mapping at adenosine stress and at rest. In addition, all HTx patients performed a maximal cardiopulmonary exercise test with gas‐exchange analysis for objective assessment of exercise capacity. Heart transplant patients had lower stress myocardial perfusion (2.9 ± 0.8 vs 3.4 ± 0.8, *p* = 0.03) and lower myocardial perfusion reserve (MPR) (2.7 ± 0.7 vs 3.8 ± 1.2, *p* < 0.001) compared to healthy controls. Furthermore, MPR in HTx patients was correlated with maximal workload (R^2^ = 0.25, *p* = 0.016), O_2_ pulse (R^2^ = 0.21, *p* = 0.026), peak O_2_ consumption (VO_2_ peak) (R^2^ = 0.25, *p* = 0.015) and O_2_ consumption (VO_2_) at the anaerobic threshold (AT) (R^2^ = 0.27, *p* = 0.011).

**Conclusion:**

Microvascular function as assessed by quantitative CMR perfusion mapping is lower in HTx patients than in healthy controls and is partly related to objective measures of exercise capacity.

## 1 INTRODUCTION

Median survival after heart transplantation (HTx) worldwide is 12.5 years (Khush et al., 2019) and the most common complication leading to death is chronic graft rejection including cardiac allograft vasculopathy (CAV) (Gjesdal et al., 2022). This condition affects coronary epicardial arteries as well as the microvasculature (Rahmani et al., 2006), and may lead to decreased myocardial blood flow. However, it should be noted that HTx patients may have lower myocardial blood flow even without epicardial CAV (Shrestha et al., 2021; Tucker et al., 2018). As diastolic function is an active process requiring oxygen (Pouleur, 1990), a low myocardial blood flow may affect cardiac pumping and consequently exercise capacity.

Quantitative cardiac magnetic resonance (CMR) perfusion imaging can be used to measure absolute myocardial perfusion and myocardial perfusion reserve (MPR) (Kellman et al., 2017), enabling assessment of coronary microvascular function without any ionizing radiation. It is therefore suitable for repeated measurements and may thus be a useful tool to follow HTx patients over time. Exercise capacity can be measured using cardiopulmonary exercise testing (CPET), which gives information on how effectively the heart, lungs and muscles deliver and use oxygen during exercise. This gives an objective measure of the patients' ability to manage their activities of daily living.

It is to our knowledge not yet known whether there is a relationship between myocardial perfusion and exercise capacity in HTx patients. Therefore, the aim of this study was to investigate if coronary microvascular function in HTx patients, as assessed by quantitative CMR perfusion mapping, differs from that in healthy controls and is related to objective measures of exercise capacity.

## 2 MATERIALS AND METHODS

### 2.1 Study population and design

The study was approved by the regional ethics committee in Lund, Sweden (Dnr 2018/948) and by the Swedish Ethical Review Authority (Dnr 2022‐02631‐01) and complies with the principles outlined in the Declaration of Helsinki. All participants gave their written informed consent before participation. HTx patients eligible for inclusion had been clinically evaluated by a cardiologist and clinically referred for CMR as a part of a post‐transplant surveillance routine. Thus, the referral was not based on suspected complications or graft rejections.

HTx patients (51 ± 15 years, 34% women) were recruited and examined between November 2019 and November 2021 at Skåne University Hospital, Lund, Sweden. Exclusion criteria included standard contraindications for CMR such as severe renal dysfunction (estimated glomerular filtration rate < 30 mL/min/1.73 m^2^), claustrophobia, pacemaker or other CMR incompatible devices. All participants were instructed to abstain from caffeine at least 24 h prior to CMR. Heart transplantation had been performed 1 year prior to the CMR in 14 HTx patients and ≥ 2 years prior to the CMR in 15 HTx patients (total range 1–26 years prior to the CMR). In total, 29 HTx patients underwent CMR followed by a maximum cardiopulmonary exercise test (CPET) on average 4 days (range 0–25 days) after CMR as a part of routine clinical follow‐up post transplantation. Twenty‐five out of 29 HTx patients also underwent coronary angiography on average 5 days (range 0–48 days) after CMR based on routine clinical follow‐up post transplantation. Three out of 29 patients underwent coronary computed tomography angiography instead of invasive coronary angiography and 1 out of 29 patients underwent invasive coronary angiography 1 year pre CMR and this data was not included for analysis.

Healthy volunteers (*n* = 26, age 57 ± 10 years, 38% females) with no cardiovascular medication, no smoking, and no history of cardiovascular or kidney disease, were included as control group from the SCAPIS study which have been described previously (Bergström et al., 2015).

### 2.2 Consent

All participants gave their written informed consent before participation.

### 2.3 CMR image acquisition

Images were acquired either on a Siemens Magnetom Aera 1.5 T (HTx *n* = 20, controls *n* = 26) or Siemens Magnetom Sola 1.5 T (HTx *n* = 9, controls *n* = 0) (Siemens Healthcare, GmbH, Erlangen, Germany). Typical image parameters are shown in Supplementary Table 1.

After acquiring localizers and scout images for definition of the left ventricular short‐axis and long‐axis (2‐, 3‐ and 4‐chamber views) planes, T2‐prepared steady‐state free precision (SSFP) short‐axis images (Kellman et al., 2017) covering the left ventricle were acquired.

Perfusion maps were obtained during adenosine stress and at rest using a single‐bolus, dual‐sequence perfusion mapping (Kellman et al., 2017) in three short‐axis views (basal, mid‐ventricular and apical) during administration of a contrast agent bolus injection (4 mL/s) of 0.05 mmol/kg (gadoteric acid, Dotarem, Guerbet, Gothia Medical AB, Billdal or Clariscan, GE Healthcare AB). The stress images were acquired after 3 min of intravenous adenosine infusion (typically 140 μg/kg/min according to clinical routine), with the infusion continued throughout image acquisition. Rest images were acquired 10 min after completion of the adenosine infusion. Heart rate, oxygen saturation and blood pressure were measured during the adenosine infusion. Quantitative perfusion and myocardial perfusion maps were generated using the Gadgetron inline perfusion mapping software (Kellman et al., 2007).

Late gadolinium enhancement (LGE) images were acquired in short‐axis covering the entire left ventricle and in the three standard long‐axis views (2‐, 3‐ and 4‐chamber), approximately 10–15 min after an intravenous administration of an additional 0.05 mmol/kg of contrast agent after rest perfusion. Late gadolinium enhancement images were used to assess presence of myocardial infarction and fibrosis.

### 2.4 CMR image analysis

All images were analyzed using the software Segment (v.3.3) (Heiberg et al., 2010). Left ventricular mass, volumes and function were quantified from manual delineations of the epicardium and endocardium of the left ventricle in short‐axis cine steady‐state free precision images by KSE (20 years of experience with CMR) and HE (25 years of experience with CMR) in consensus. Presence of infarction and fibrosis was assessed by visual evaluation of hyper‐enhancement on LGE‐images by KD (3 years of experience with CMR) and HE in consensus. Presence of edema was assessed by visual evaluation of increased signal intensity on T2 weighted images by KD and HE in consensus.

The endo‐ and epicardial borders for the short‐axis perfusion maps were manually delineated at stress and rest by KD and reviewed by HE. The delineations were kept approximately two pixels away from the endo‐ and epicardial borders to avoid inclusion of blood pool or extracardiac structures within the myocardial contours. Image artifacts were excluded from the regions of interest. Myocardial perfusion in ml/min/g was evaluated regionally according to the 17‐segment model (Cerqueira et al., 2002), excluding the apical segment, therefore resulting in 16 segments per patient. Myocardial perfusion was then calculated globally (average myocardial perfusion for all 16 segments). Segments with regional hypoperfusion corresponding to a coronary artery stenosis supplying that region were excluded from the analysis of global myocardial perfusion. LGE‐positive segments due to infarction/fibrosis were also excluded. Perfusion inhomogeneity was assessed visually and graded into three groups (homogenous perfusion, moderate inhomogeneity, and severe inhomogeneity) according to a previous study (Wenning et al., 2015). Myocardial perfusion reserve was calculated as the ratio between myocardial perfusion during stress and at rest and was calculated both regionally and globally as described above. Since myocardial perfusion at rest is related to the rate‐pressure product (RPP), the resting myocardial perfusion in each subject was reported corrected for RPP as follows:

Corrected myocardial perfusion at rest=(myocardial perfusion at rest×mean group RPP)/(individual RPP)

as previously described (Uren et al., 1994).

Mean group RPP was determined in both HTx patients and healthy controls. Rate‐pressure product corrected MPR was calculated as

Rate‐pressure product corrected MPR=myocardial perfusion at stress/corrected myocardial perfusion at rest

### 2.5 Coronary angiography

Coronary angiography was performed on 25 HTx patients according to clinical routine by an experienced angiographer. Each of the three vessels (LAD, RCA and LCX) were examined. Cardiac allograft vasculopathy severity was classified according to the International Society for Heart and Lung Transplantation nomenclature (Mehra et al., 2010), where CAV 0 = not significant, CAV 1 = mild, CAV 2 = moderate and CAV 3 = severe.

### 2.6 Cardiopulmonary exercise test

Cardiopulmonary exercise tests were performed on a cycle ergometer according to the Swedish Board for Accreditation and Conformity Assessment (SWEDAC)‐accredited method used at the Department of Clinical Physiology, Skåne University Hospital, Lund. Resting blood pressure and ECG were acquired before the test. The starting workload (in watt) and the gradient of the ramp (the increase in watt per minute) were decided based on sex, age and self‐estimated fitness level. The exercise test ended when the patient could not keep up an even pace due to exhaustion. A respiratory exchange ratio ≥ 1.10 for subjects aged twenty to forty‐nine and ≥ 1.05 for subjects aged 50 and older was considered a maximal test (Edvardsen et al., 2014). The exercise test was examined by an experienced physician blinded to CMR and coronary angiography results. Ventilatory anaerobic threshold (AT) was detected by combining the V‐slope, ventilatory equivalent for O_2_ and end‐tidal methods as previously described (Balady et al., 2010). Maximal workload (watt), O_2_ pulse (mL/min), VO_2_ peak (mL/min), VO_2_ at AT (mL/min) and the V_E_/VCO_2_‐slope were determined.

### 2.7 Statistical analysis

Statistical analysis was performed using the software Graph Pad Prism version 9.3.1 (Graph Pad Software Inc., La Jolla, CA, USA). Normal distribution was tested by visual inspection and by using Shapiro‐Wilk test. Results are presented as mean ± SD or as median and interquartile range (Q1,Q3) as appropriate. Mean values were compared using an unpaired t‐test and median values were compared using the Mann‐Whitney U‐test. The relationships between continuous variables were assessed with linear regression and presented as R^2^. Univariable linear regression analysis was performed to show potential associations between VO_2_peak and age, sex, heart rate, systolic blood pressure and MPR. These factors were selected based on clinical relevance as factors that are potentially associated with VO_2_peak and MPR. Differences with *p* < 0.05 were considered statistically significant.

## 3 RESULTS

Twenty‐nine HTx patients, referred for clinical CMR, were included in the study. Stress perfusion images were not assessable in two cases due to respiratory motion artifacts. In two cases no splenic switch‐off sign was observed during adenosine stress and no increase perfusion was accomplished and these two cases were therefore excluded from the stress perfusion analysis. Rest perfusion images were not assessable in two cases due to respiratory motion artifacts. A stress perfusion image in one healthy control was not assessable due to respiratory motion artifact.

Baseline characteristics of the study cohort (*n* = 29) are shown in Table 1. HTx patients and healthy controls were similar in respect to age and sex. However, HTx patients had a lower ejection fraction compared to healthy controls.

**Table 1:** Patient characteristics.

	HTx patients (*n* = 29)	Healthy controls (*n* = 26)
Female sex	34% (10/29)	38% (10/26)
Age, years	53 (35,63)	61 (50,64)
Year after transplant	5.3 ± 6.3	n/a
Prior ACR ≥ 2 R	10% (3/29)	n/a
Prior AMR	3% (1/29)	n/a
Current ACR ≥ 2 R	0% (0/14)	n/a
Current AMR	0% (0/14)	n/a
Betablocker	21% (6/29)	n/a
Immunosuppression	100% (29/29)	n/a
Prednisolon	72% (21/29)	
Tacrolimus	83% (24/29)	
Sandimmun	7% (2/29)	
Everolimus	21% (6/29)	
Mycophenolate mofetil	86% (25/29)	
Azathioprine	7% (2/29)	
BSA, m^2^	2.0 ± 0.2	2.0 ± 0.2
LVM, mL	97 ± 17	91 ± 28
LVM, g	102 ± 17	96 ± 29
LVMI, g/m^2^	52 ± 8	48 ± 16
LVEDV, mL	155 ± 30	158 ± 35
LVEDVI, mL/m^2^	79 ± 14	79 ± 19
LVESV, mL	67 ± 18	61 ± 18
LVESVI, mL/m^2^	34 ± 9	30 ± 10
LVSV, mL	89 (75,99)	95 (83,103)
LVSVI, mL/m^2^	45 ± 9	48 ± 11
LVEF, %	58 (51,62)*	62 (59,64)
CO, l/min	7.1 ± 1.5	6.7 ± 1.9
CI, l/min	3.7 ± 0.1	3.3 ± 0.9

*Note*: Data presented as percent or mean ± SD or median (IQR).

Abbreviations: ACR, acute cellular rejection; AMR, anti‐body mediated rejection; BSA, body surface area; CI, cardiac index; CO, cardiac output; EDV, end diastolic volume; EF, ejection fraction; ESV, end systolic volume; g, grams; g/m^2^, grams per square meter; HTx, heart transplant; LVM, left ventricular mass; LVMI, left ventricular mass index;mL, milliliters; mL/m^2^, milliliters per square meter; l/min, liters per minute; SV, stroke volume.

*p < 0.05 for differences between the groups (Mann‐Whitney U test).

Five HTx patients had hyper‐enhancement on LGE images due to infarction (*n* = 2) or midmural fibrosis (*n* = 3). One patient had a slightly increased signal on T2‐weighted images. However, this patient had no clinical or biopsy‐verified signs of acute rejection at the time of the CMR examination (Table 2). The remaining HTx patients (*n* = 28) had no increased signal on T2‐weighted images. Out of 25 coronary angiographies one patient was classified as CAV 1 (myocardial perfusion at stress 2.42, MPR 1.94), one patient was classified as CAV 2 (myocardial perfusion at stress 1.91, MPR 2.08) and one patient was classified as CAV 3 (myocardial perfusion at stress 0.61, MPR 1.22), showing a significant left main stenosis with corresponding regional stress‐induced ischemia (Table 2). The patient classified as CAV 3 was one of the two cases with no splenic switch‐off sign and was therefore not included in the perfusion analysis. The remaining HTx patients were classified as CAV 0.

**Table 2:** Cardiac magnetic resonance and coronary angiography findings.

	All (*n* = 29)	Females (*n* = 10)	Males (*n* = 19)
LGE+	17% (5/29)	20% (2/10)	16% (3/19)
Infarction	7% (2/29)	10% (1/10)	5% (1/19)
Non‐infarction	10% (3/29)	10% (1/10)	11% (2/19)
Regional perfusion deficit	24% (6/25)*	11% (1/9)	31% (5/16)
Homogeneous perfusion	36% (9/25)*	33% (3/9)	38% (6/16)
Inhomogeneous perfusion	64% (16/25)*	67% (6/9)	63% (10/16)
Myocardial edema	3% (1/29)	0% (0/10)	5% (1/19)
Coronary angiography	86% (25/29)	80% (8/10)	89% (17/19)
Obstructive epicardial CAD	4% (1/28)†	0% (0/9)	5% (1/19)
ISHLT CAV_0_	79% (22/28)†	78% (7/9)	79% (15/19)
ISHLT CAV_1_	4% (1/28)†	11% (1/9)	0% (0/19)
ISHLT CAV_2_	4% (1/28)†	0% (0/9)	5% (1/19)
ISHLT CAV_3_	3% (1/28)	0% (0/10)	5% (1/19)

*Note*: Data presented as percent of HTx patients in each group. Missing data: *: *n* = 4 HTx patients were excluded from the perfusion analysis due to respiratory motion artifacts (*n* = 2) and no splenic switch‐off sign (*n* = 2). †: *n* = 1 HTx patient was excluded from the analysis due to no available current invasive or CT angiogoprahy.

Abbreviations: CAD, coronary artery disease; CAV, cardiac allograft vasculopathy; ISHLT, the International Society for Heart and Lung Transplantation; LGE, late gadolinium enhancement.

### 3.1 Myocardial perfusion

**Figure 1:**
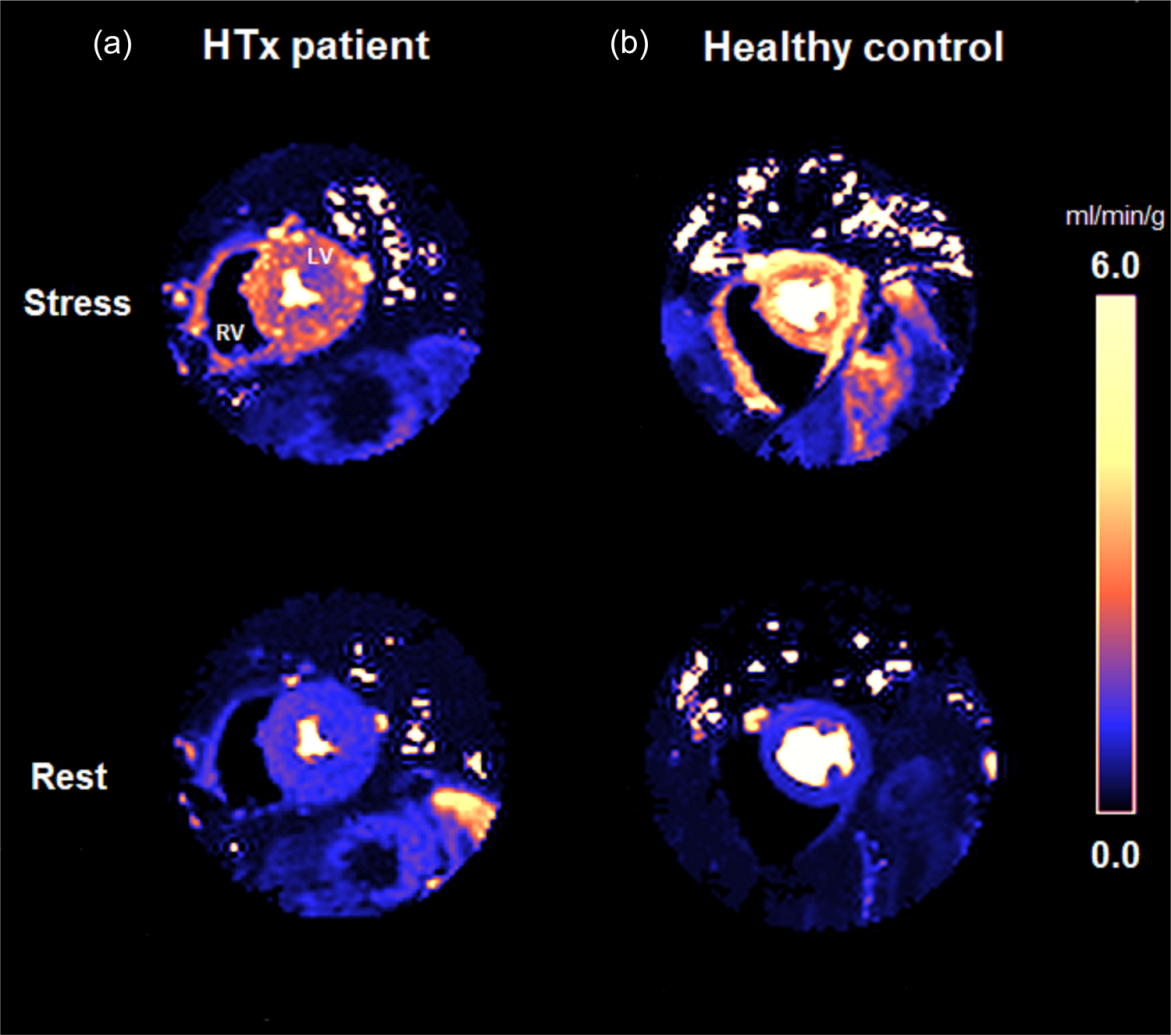
Example of quantitative myocardial perfusion in a heart transplant (HTx) patient and a healthy control. Quantitative short‐axis mid‐ventricular myocardial perfusion maps at adenosine stress and at rest in A) a 61‐year‐old HTx patient and B) a 61‐year‐old healthy control. Both myocardial perfusion and myocardial perfusion reserve were lower in the HTx patient compared to the healthy control. The colour scale represents myocardial perfusion expressed in absolute values (yellow= high myocardial perfusion, blue= low myocardial perfusion). Abbreviations: HTx, heart transplant; LV, left ventricle; RV, right ventricle.

**Figure 2:**
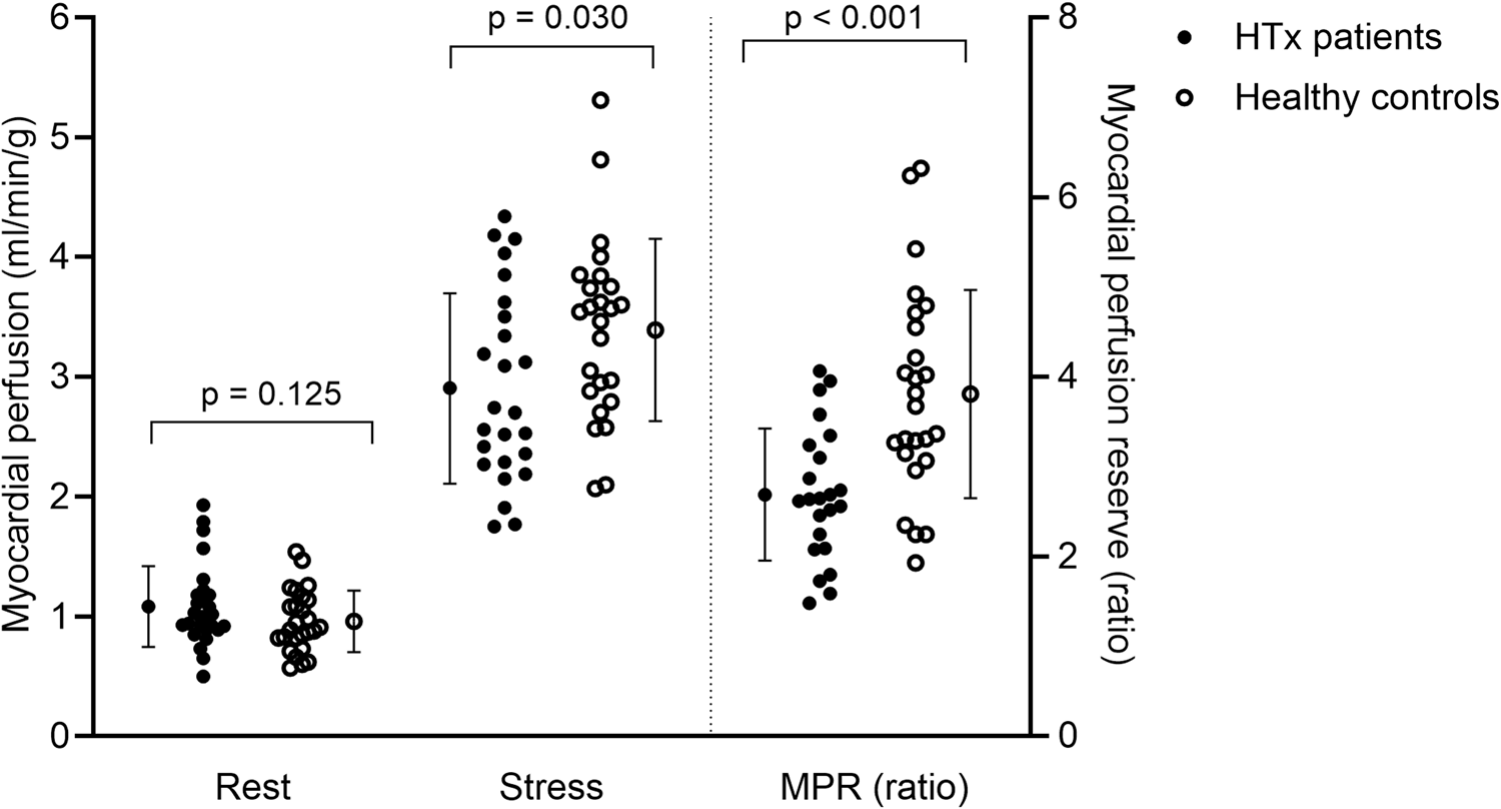
Myocardial perfusion at rest, stress and myocardial perfusion reserve (MPR) in HTx patients compared to healthy controls. HTx patients had a lower myocardial perfusion at stress and a lower MPR compared to healthy controls. Error bars represent mean ± 1 SD. Mean values were compared using an unpaired t‐test. Rest: HTx *n* = 27, controls *n* = 26. Stress: HTx *n* = 25, controls *n* = 25. MPR: HTx *n* = 23, controls *n* = 25. Abbreviations: HTx, heart transplant; MPR, myocardial perfusion reserve.

Figure 1 shows examples of quantitative short‐axis myocardial perfusion maps in a HTx patient and a healthy control. Segmentally quantified myocardial blood flow values corresponding to the perfusion maps are presented in Supplemental Table 2. Myocardial perfusion at stress for the HTx patients was lower than healthy controls (2.9 ± 0.8 vs 3.4 ± 0.8, *p* = 0.030) whereas there was no significant difference at rest (1.1 ± 0.3 vs 0.96 ± 0.3, *p* = 0.125). Furthermore, MPR in HTx patients was significantly lower than in healthy controls (2.7 ± 0.7 vs 3.8 ± 1.2, *p* < 0.001; Figure 2).

Stress myocardial perfusion was not different between HTx patients who had had heart transplantation 1 year before and ≥ 2 years before (3.0 ± 0.8 vs 2.8 ± 0.8, *p* = 0.530). Myocardial perfusion values were the same when LGE‐positive segments were included.

Inhomogeneous myocardial perfusion was observed in sixteen (64%) HTx patients (Table 2). Of these, five (20%) had moderate inhomogeneous perfusion while eleven (44%) had severe inhomogeneous perfusion.

As shown in Supplementary Figure 1, there was no significant difference in myocardial perfusion between female and male HTx patients (rest 1.2 ± 0.3 vs 1.0 ± 0.4, *p* = 0.368; stress 3.2 ± 0.8 vs 2.8 ± 0.8, *p* = 0.257). There was no significant difference in myocardial perfusion between female and male healthy controls (rest 1.0 ± 0.2 vs 0.9 ± 0.3, *p* = 0.329; stress 3.5 ± 0.7 vs 3.3 ± 0.8, *p* = 0.741).

### 3.2 Myocardial perfusion in relation to hemodynamics and CPET values

Results from analyses of hemodynamic variables are shown in Table 3. The rate‐pressure product (RPP) and heart rate at rest was higher in HTx patients than healthy controls (9740 ± 2088 vs 7687 ± 1803, *p* < 0.001 and 80 ± 11 vs 64 ± 10 bpm, *p* < 0.001). No significant difference in RPP and heart rate was found between HTx patients and controls during stress (*p* = 0.172 and *p* = 0.400). Corrected MPR for RPP was significantly lower in HTx patients than in healthy controls (2.6 ± 0.6 vs 3.7 ± 1.2, *p* < 0.001).

**Table 3:** Hemodynamics and myocardial perfusion.

	HTx patients	Healthy controls	*p* value
Rest HR, bpm	80 ± 11	64 ± 10	<0.001
Stress HR, bpm	87 ± 13	90 ± 16	0.400
Rest systolic BP, mmHg	122 ± 15	119 ± 17	0.495
Stress systolic BP, mmHg	116 ± 16	122 ± 16	0.179
Rest RPP	9740 ± 2088	7687 ± 1803	<0.001
Stress RPP	10155 ± 2041	10995 ± 2365	0.172
Rest MP, mL/min/g	1.1 ± 0.3	0.96 ± 0.3	0.125
Rest MP (RPP adjusted), mL/min/g	1.1 ± 0.3	0.97 ± 0.2	0.134
Stress MP, mL/min/g	2.9 ± 0.8	3.4 ± 0.8	0.030
MPR	2.7 ± 0.7	3.8 ± 1.2	<0.001
MPR (RPP adjusted)	2.6 ± 0.6	3.7 ± 1.2	<0.001

*Note*: Data presented as mean ± SD.

Abbreviations: BP, blood pressure; bpm, beats per minute; HTx, heart transplant, HR, heart rate; mmHg, millimeters of mercury; mL/min/g, millimeters per minute per gram; MP, myocardial perfusion; MPR, myocardial perfusion reserve; RPP, rate‐pressure product.

Myocardial perfusion at rest was correlated with RPP at rest (R^2^ = 0.35, *p* = 0.001) and heart rate at rest (R^2^ = 0.27, *p* = 0.007). There was no correlation between myocardial perfusion at rest and systolic blood pressure (R^2^ = 0.11, *p* = 0.091; Supplementary Figure 2). Furthermore, myocardial perfusion was not correlated with stroke volume (R^2^ = 0.003, *p* = 0.786).

**Figure 3:**
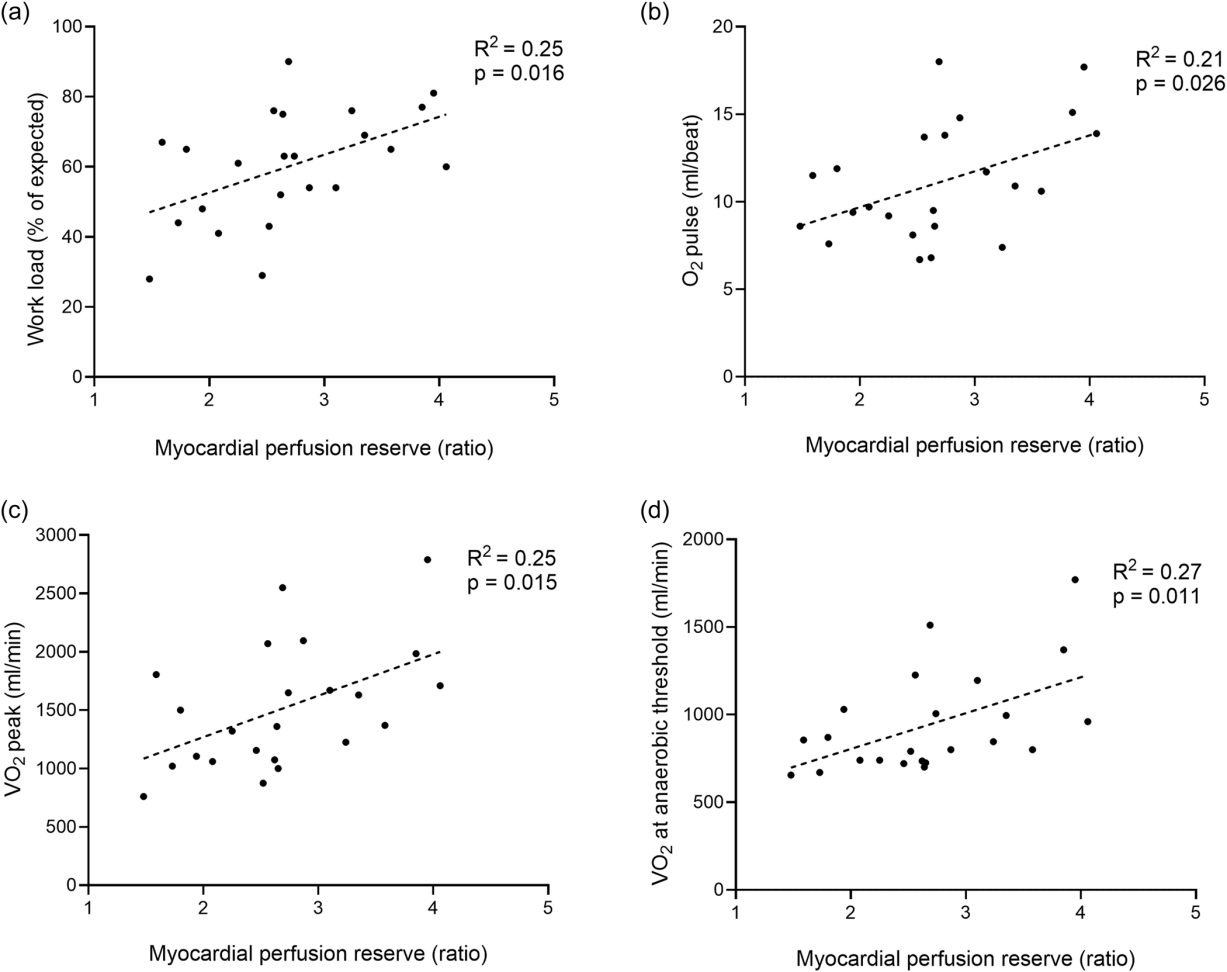
The relationship between objective measures of exercise capacity and myocardial perfusion reserve (MPR). MPR was correlated with A) maximal workload, B) O_2_ pulse, C) VO_2_ peak and D) VO_2_ at anaerobic threshold in HTx patients (*n* = 23). The dashed line represents the line of regression. The relationship was assessed with simple linear regression and presented as R^2^.

Myocardial perfusion reserve in HTx patients was correlated with maximal workload (R^2^ = 0.25, *p* = 0.016), VO_2_ peak (R^2^ = 0.25, *p* = 0.015), VO_2_ at AT (R^2^ = 0.27, *p* = 0.011), and O_2_ pulse (R^2^ = 0.21, *p* = 0.026) (Figure 3). Cardiopulmonary exercise test variables including maximal workload, peak heart rate, VO_2_ peak, VO_2_ at AT, V_E_/VCO_2_‐slope and O_2_ pulse are shown in Table 4. There was no correlation between myocardial perfusion at stress and any CPET value (Supplementary Figure 3). Univariable regression analysis (Supplementary Table 3) showed that female sex was associated with a lower VO_2_peak.

**Table 4:** Cardiopulmonary exercise test results in HTx patients.

	All	Females	Males
Work load, watt	119 ± 47	100 ± 30	130 ± 51
Work load, % of expected	61 ± 15	63 ± 12	59 ± 17
VO_2_ peak, mL/min	1500 ± 475	1265 ± 245	1620 ± 525
VO_2_ peak, mL/kg/min	18.7 ± 4.6	17.5 ± 3.9	19.4 ± 4.9
VO_2_ at AT, mL/min	870 (740,1008)	823 (733,916)	910 (740,1195)
VO_2_ at AT, mL/kg/min	10.6 (9.4,14.2)	11.3 (10.2,13.4)	10.6 (9.2,14.9)
V_E_/VCO_2_‐slope	31 ± 8	31 ± 5	31 ± 9
O_2_ pulse, mL/beat	11.2 ± 3.0	9.3 ± 1.9	12.2 ± 3.0*

*Note*: Data presented as mean ± SD or mean (IQR).

Abbreviations: AT, anaerobic threshold; bpm, beats per minute; mL, milliliters; mL/min, milliliters per minutes; mL/kg/min; milliliters per kilograms per minute; VE, minute ventilation; VCO2, carbon dioxide output; VO2, oxygen uptake.

*p < 0.05 for differences between the sexes (unpaired t‐test).

## 4 DISCUSSION

This study is the first to investigate coronary microvascular function in HTx patients using fully quantitative perfusion CMR in relation to objective measures of exercise capacity. Results showed that HTx patients had a lower myocardial perfusion at stress and a lower MPR than healthy volunteers. Also, MPR correlated with measures of exercise capacity in HTx patients.

There was a positive correlation between MPR and objective measures of exercise capacity (maximal workload, VO_2_ peak, VO_2_ at AT, and O_2_ pulse), whereas myocardial perfusion at stress did not correlate with exercise capacity parameters. Myocardial perfusion reserve has previously been shown to be a stronger predictor of all‐cause mortality than myocardial perfusion at stress in patients with coronary artery disease (Knott et al., 2020). Thus, MPR probably carries different prognostic information than myocardial perfusion at stress alone. Myocardial perfusion reserve can be said to mirror the ability of the heart to increase its vasodilator‐ and pumping capacity when needed, and this may explain its correlation to VO_2_ peak. A reduced MPR could therefore represent a central limitation to exercise capacity, potentially related to microvascular dysfunction or early graft dysfunction. While the correlation between MPR and VO_2_ peak was statistically significant (R^2^ = 0.25, *p* = 0.015), 75% of the variance in the peak VO_2_ is still explained by other factors than MPR. Thus, the use of MPR alone to predict VO_2_ peak for a specific patient is limited and the relationship between MPR and exercise capacity is likely multifactorial.

There are several non‐coronary and peripheral factors that could also influence VO_2_peak after heart transplantation, including haemoglobin level (Webb et al., 2023), steroid use (Renlund et al., 1996), endothelial dysfunction (Hermann et al., 2011) and sex related differences (Leung et al., 2003). Previous studies further indicate that the reduced VO_2_peak in HTx patients is driven by both central and peripheral mechanisms such as skeletal muscle deconditioning (Foulkes et al., 2023; Kao et al., 1994). Therefore, a combined assessment of MPR and exercise capacity could provide complementary information about central and peripheral contributors to reduced functional capacity after transplantation. In patients with low exercise capacity, reduced MPR could suggest a central or microvascular component, whereas a normal MPR may point toward a peripheral limitation. Since most patients had no angiographic evidence of CAV, evaluating myocardial perfusion in relation to exercise capacity in this early phase after transplantation is important. This could identify early graft dysfunction before the development of established CAV at a stage when intervention is still possible. However, larger longitudinal studies are needed to determine whether impaired MPR, alone or in combination with measures of exercise capacity, predicts graft dysfunction and development of CAV.

These findings support the view that both central and peripheral mechanisms should be targeted to improve exercise capacity. Targeting MPR and microvascular dysfunction for new post‐transplantation therapies could potentially benefit selected patients. However, based on the associations observed in this study, a combined strategy addressing both MPR and peripheral factors is likely most effective.

Low myocardial perfusion at stress can be caused by hypoperfusion due to both obstructive CAD or microvascular dysfunction (Rahman et al., 2019; Salerno and Beller, 2009). In HTx patients, low myocardial perfusion has been associated with CAV, a diffuse disease of the epicardial coronary arteries and the coronary microcirculation (Shrestha et al., 2021). In this study, obstructive CAD was only found in one patient (significant left main stenosis) and these stress images were excluded from the analysis due to no splenic switch‐off sign. Thus, the low myocardial perfusion compared to healthy volunteers is likely caused by impaired microvascular function. The observed low myocardial perfusion primarily followed an inhomogenous flow reduction in the myocardium, as 64% of HTx patients showed a segmental flow reduction. This could be of clinical relevance, as one previous study (Wenning et al., 2015) suggested that HTx patients with inhomogeneous myocardial perfusion may be at greater risk for developing allograft dysfunction than those with a homogeneous perfusion reduction. Also, this finding supports the idea that microvascular dysfunction can potentially occur regionally in the myocardium. However, further follow‐up studies are needed to evaluate the prognostic information of perfusion inhomogeneity beyond absolute perfusion and MPR.

Assessment of microvascular function provides prognostic information for HTx patients (Clerkin et al., 2022), and MPR has been suggested as a non‐invasive diagnostic tool for early detection of microvascular pathology (Chih et al., 2016). Current guidelines include cardiac positron emission tomography (PET) as a non‐invasive option for quantitative assessment of myocardial perfusion in HTx patients (Chih et al., 2020). However, a recent multicenter study showed that fully quantitative perfusion CMR can discriminate significant CAV (Jablonowski et al., 2025), supporting the relevance of quantitative CMR as a non‐invasive option for HTx surveillance and for detecting CAV.

Nearly half of the HTx patients in the current study were examined 1 year after heart transplantation, however on group level the mean follow up time was 5.3 ± 6.3 years. Stress myocardial perfusion (2.9 mL/min/g) and the resulting MPR (2.7) in the present study were higher than previously published data (Bravo et al., 2018; Miller et al., 2014) which may be explained by differences in time to follow‐up. Bravo et al. had a mean follow up time of 12 years and demonstrated a myocardial perfusion value of 1.8 ± 0.50 mL/min/g (Bravo et al., 2018) and Miller et al. with a median follow up time of 7 years showed a myocardial perfusion value of 1.5 ± 0.49 mL/min/g (Miller et al., 2014). Follow‐up time is an important factor to consider when measuring myocardial perfusion in HTx as CAV is a progressive condition that lowers the myocardial perfusion over time. The prevalence of CAV increases beyond the first year after surgery, thus, CAV may not yet have developed in many of the HTx patients in the present study. This is supported by the coronary angiography findings, where 22 out of 25 HTx patients were classified as CAV 0. In line with the current results, Shresta et al. showed that HTx patients with non‐CAV had an average myocardial perfusion of 2.7 mL/min/g using quantitative PET (Shrestha et al., 2021), which corresponds well to our finding (2.9 mL/min/g). The low prevalence of CAV and the high myocardial perfusion suggest that the patient group of the present study may be healthier than previous studies (Bravo et al., 2018; Miller et al., 2014).

Resting heart rate was significantly higher in HTx patients than in healthy controls, but no difference was found between the two groups for resting myocardial perfusion. Previous studies suggest that myocardial perfusion at rest is higher in HTx patients than in healthy controls (Schwitter et al., 2000). This may be explained by loss of parasympathetic control of the sinus node in the transplanted heart which results in elevated heart rate at rest. Moreover, it could be speculated that there is variability in underlying inflammation in the allografts due to acute rejection in the early years after transplant. The pro‐inflammatory state could potentially increase the myocardial perfusion at rest. However, none of the HTx patients that underwent CMR 1 year post transplant (*n* = 14) had signs of biopsy‐verified acute rejection. Thus, inflammation due to acute rejection is not likely to have developed in our study cohort, possibly explaining the lack of difference in myocardial perfusion at rest compared to previous studies (Schwitter et al., 2000).

Previous studies in healthy populations have shown that females have higher myocardial perfusion at stress and rest than males, suggesting that sex is a contributing factor to myocardial perfusion (Nickander et al., 2020). In this study there was no statistically significant differences in the perfusion between female and male HTx patients (Supplementary figure 1). The lack of observed sex difference in HTx patients is most probably due to the limited number of patients (10 females). However, there is a possibility that sex might not contribute as much to myocardial perfusion in HTx patients as in healthy individuals and therefore more studies are needed to confirm or reject this finding.

Even though rest and stress perfusion numerically were slightly higher in female controls compared to male controls, these differences were not statistically significant as opposed to previous findings (Nickander et al., 2020). In addition to a limited number of participants, one possible explanation could be the significant age difference among female and male controls. In our cohort, female controls were older than male controls with a mean age of 62 years compared with 54 years in male controls.

In addition to the quantitative assessment of myocardial perfusion both regionally and globally, this study has highlighted the additional diagnostic benefits of CMR. Tissue characterization using T2‐weighted images in combination with LGE‐imaging, enabled the detection of edema, fibrosis, and infarction. Thus, CMR provides complementary information within a single examination and could be a valuable non‐invasive complement to already established methods such as coronary angiography and echocardiography in the assessment of HTx patients.

### 4.1 Limitations

The study cohort was relatively small (*n* = 29), and the number of female HTx patients (*n* = 10) was half of the number of male HTx patients (*n* = 19). This results in limited power when showing differences between the groups. Moreover, imaging with intravascular ultrasound or optical coherence tomography is considered the gold standard and is a more sensitive measure of CAV. Only half of HTx patients in our study underwent any kind of intravascular imaging procedure. Likewise, invasive measurements such as index of microcirculatory resistance and coronary flow reserve could give more information on the microcirculation in HTx patients but were not performed as part of the clinical follow‐up angiography. In addition, the HTx physiology with denervation and chronotropic incompetence might make conventional pharmacological stress (adenosine) less reliable.

## 5 CONCLUSIONS

Microvascular function as assessed by quantitative CMR perfusion mapping is lower in HTx patients than healthy controls. Furthermore, MPR is related to objective measures of exercise capacity such as VO_2_ peak in HTx patients.

## AUTHOR CONTRIBUTIONS

Kristian Dimovski, Grunde Gjesdal, Katarina Steding‐Ehrenborg, Oscar Braun, Håkan Arheden, and Henrik Engblom, contributed to the conceptualization of the study. Robert Jablonowski, Hui Xue, and Peter Kellman contributed to data analysis. Kristian Dimovski, Robert Jablonowski, and Henrik Engblom conducted the formal analysis. Kristian Dimovski, Katarina Steding‐Ehrenborg, Peter Kellman, Oscar Braun, Håkan Arheden, and Henrik Engblom participated in the interpretation of data. Håkan Arheden, and Henrik Engblom, provided fundings. Kristian Dimovski wrote the original draft. All authors revised, gave critically intellectual input, and approved the final version of the manuscript.

## CONFLICTS OF INTEREST STATEMENT

The authors declare no conflicts of interest.

## Supporting information

Supporting File 1

Supporting File 2

Supporting File 3

Supporting File 4

## Data Availability

The datasets generated during and analyzed during the current study are available from the corresponding author on reasonable request. The data that support the findings of this study are available on request from the corresponding author. The data are not publicly available due to privacy or ethical restrictions.
