# Supplementary figures and images for: Myocardial perfusion reserve is low in heart transplant patients and is related to exercise capacity
Source: Clin Physiol Funct Imaging. 2026 Jul 16;46(4):e70082. doi: 10.1111/cpf.70082 (PMC13374594; doi:10.1111/cpf.70082)

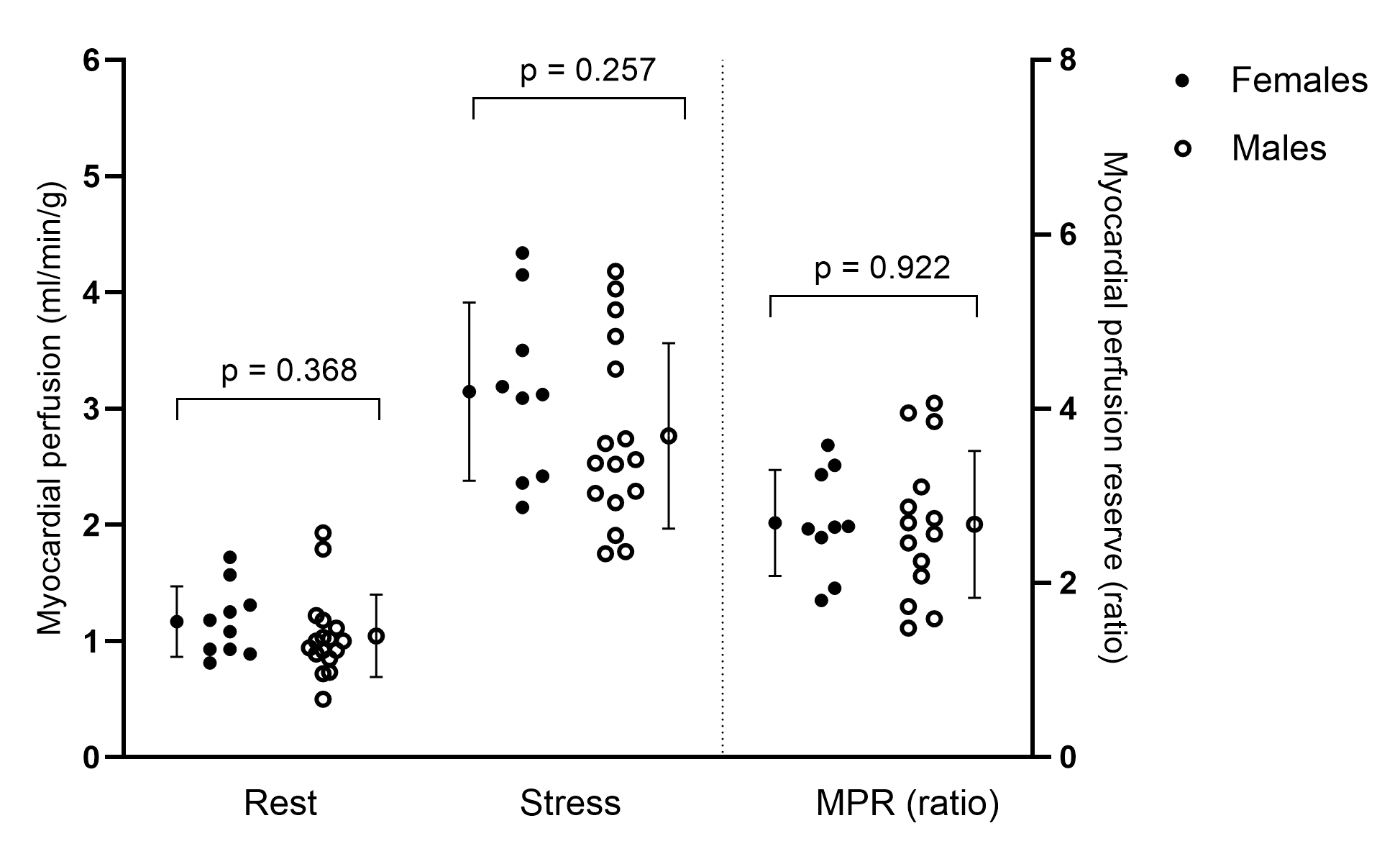

Supplement: Supplementary file 1 — Supporting File 1 [file CPF-46-0-s001.tif]

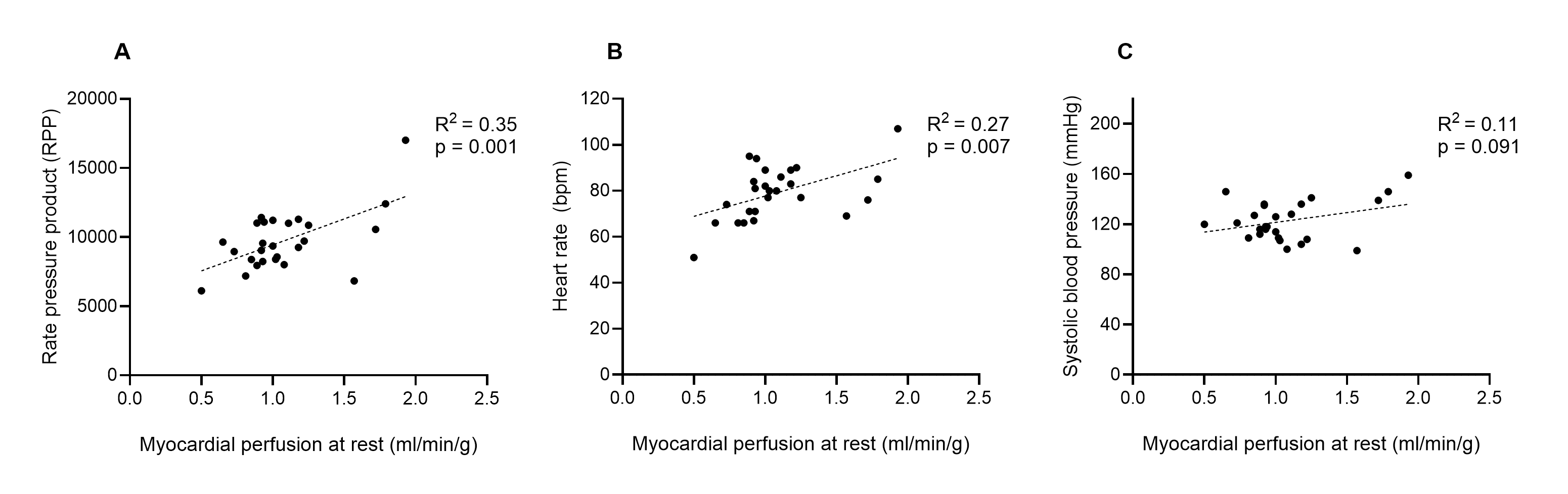

Supplement: Supplementary file 2 — Supporting File 2 [file CPF-46-0-s003.tif]

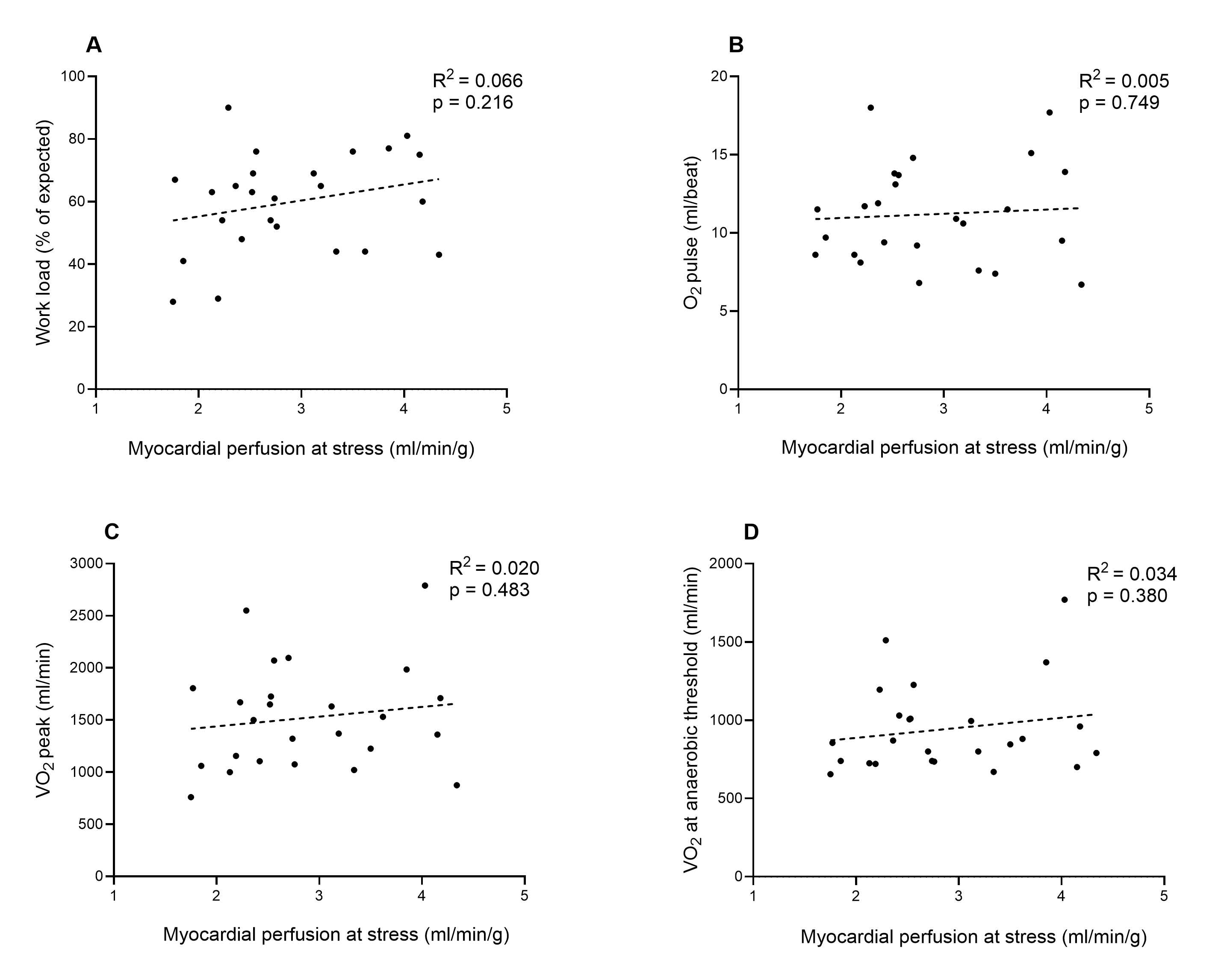

Supplement: Supplementary file 3 — Supporting File 3 [file CPF-46-0-s004.tif]
