# Supplementary material for: Myocardial perfusion reserve is low in heart transplant patients and is related to exercise capacity
Source: Clin Physiol Funct Imaging. 2026 Jul 16;46(4):e70082. doi: 10.1111/cpf.70082 (PMC13374594; doi:10.1111/cpf.70082)
Supplement: Supplementary file 4 — Supporting File 4 [file CPF-46-0-s002.docx]

**Supplementary Material**

**Supplemental Figure 1. Sex differences in myocardial perfusion and myocardial perfusion reserve in heart transplant patients.** There was no significant difference in myocardial perfusion at rest and at stress between females (filled circles) and males (open circles). Error bars represent mean ± 1SD. Mean values were compared using an unpaired t-test.

Abbreviations: MPR, myocardial perfusion reserve

**Supplemental Figure 2. Correlation between myocardial perfusion at rest and hemodynamic parameters.** Myocardial perfusion was correlated with A) rate pressure product (RPP) and B) heart rate. There was no correlation with C) systolic blood pressure. The dashed line represents the line of regression. The relationship was assessed with simple linear regression and presented as R^2^.

**Supplemental Figure 3.** Linear correlation between myocardial perfusion at stress and A) workload, B) O_2_ pulse, C) VO_2_ peak and D) VO_2_ at anaerobic threshold. No statistically significant correlations were found.

**Supplemental Table 1.** Typical CMR image parameter

| Sequence parameters | **Cine imaging**  Siemens Sola | **Cine imaging**  Siemens Aera | **LGE**  Siemens Sola | **LGE**  Siemens Aera |
| --- | --- | --- | --- | --- |
| Repetition time, ms | 2.7 | 2.7 | Individual | Individual |
| Echo time, ms | 1.11 | 1.16 | 1.18 | 1.13 |
| Flip angle, degrees | 53 | 69 | 50 | 50 |
| Acquired spatial res, mm | 1.8 x 1.8 x 8 | 1.8 x 1.8 x 8 | 1.7 x 1.7 x 8 | 1.7 x 1.7 x 8 |
| Reconstructed spatial res, mm | 1.8 x 1.8 x 8 | 1 x 1 x 8 | 1.4 x 1.4 x 8 | 1.6 x 1.6 x 8 |
| Reconstructed temporal res, ms | 34 | 32 | n/a | n/a |
| Reconstructed time phases | 25 | 25 | n/a | n/a |
| Parallell imaging factor | 3 | 3 | 2 | 2 |
| Slice gap, mm | 0 | 0 | 0 | 0 |

Abbreviations: CMR, Cardiac Magnetic Resonance; LGE, Late Gadolinium Enhancement

**Supplemental Table 2.** Segmental myocardial blood flow values for the HTx patient and the healthy control shown in Figure 1.

| **Segment** | HTx patient | | Healthy control | |
| --- | --- | --- | --- | --- |
|  | **Stress flow (ml/min/g)** | **Rest flow (ml/min/g)** | **Stress flow (ml/min/g)** | **Rest flow (ml/min/g)** |
| 1 Basal anterior | 2.62 | 1.37 | 4.40 | 1.02 |
| 2 Basal anteroseptal | n/a | n/a | n/a | 0.78 |
| 3 Basal inferoseptal | 2.15 | 1.05 | 2.43 | 0.81 |
| 4 Basal inferior | 2.31 | 0.74 | 3.88 | 0.77 |
| 5 Basal inferolateral | 2.60 | 0.88 | 3.80 | 0.71 |
| 6 Basal anterolateral | 2.54 | 1.23 | 4.82 | 0.92 |
| 7 Mid anterior | 2.08 | 1.27 | 3.46 | 1.14 |
| 8 Mid anteroseptal | 2.66 | 1.32 | 3.37 | 0.85 |
| 9 Mid inferoseptal | 2.25 | 1.20 | 2.91 | 0.78 |
| 10 Mid inferior | 2.23 | 1.07 | 3.38 | 0.83 |
| 11 Mid inferolateral | 2.08 | 1.14 | 3.56 | 0.77 |
| 12 Mid anterolateral | 2.16 | 1.12 | 3.23 | 0.93 |
| 13 Apical anterior | 2.96 | 1.51 | 4.21 | 1.05 |
| 14 Apical septal | 2.93 | 1.35 | 3.32 | 0.98 |
| 15 Apical inferior | 2.39 | 1.00 | 3.22 | 0.95 |
| 16 Apical lateral | n/a | 1.12 | 3.65 | 0.87 |
| GLOBAL | 2.42 | 1.16 | 3.58 | 0.89 |

Abbreviations: HTx, heart transplant; n/a, not available

Segmental values represent absolute myocardial blood flow in ml/min/g. The global value represents mean myocardial blood flow for all evaluable segments. Segments 2 and 16 were excluded due to artifacts.

**Supplemental Table 3.** Univariable analysis for predicted VO2 peak and associated variables in HTx patients

|  | **Estimate** | **95% CI (asymptotic)** | ***P* value** |
| --- | --- | --- | --- |
| **VO_2_ peak** |  |  |  |
| Age | -12 | -28 to 4.6 | 0.15 |
| Sex[female] | -451 | -884 to -18 | **0.04** |
| Heart rate (rest) | -8.7 | -31 to 14 | 0.43 |
| Systolic blood pressure (rest) | -7.7 | -23 to 7.7 | 0.31 |
| MPR | 355 | 76 to 633 | **0.015** |

Abbreviations: VO_2_, Oxygen uptake; MPR, myocardial perfusion reserve
